# Integrating AI tools in teacher professional learning: a conceptual model and illustrative case

**DOI:** 10.3389/frai.2023.1255089

**Published:** 2023-12-07

**Authors:** Kairit Tammets, Tobias Ley

**Affiliations:** ^1^Center for Educational Technology, Tallinn University, Tallinn, Estonia; ^2^Center for Digitalization in Lifelong Learning, University for Continuing Education Krems, Krems an der Donau, Austria

**Keywords:** artificial intelligence, learning analytics, teacher professional learning, professional vision, co-design

## Abstract

This conceptual paper aims to explore the complex nature of integrating AI technologies in teacher professional learning, highlighting the potential for AI to synergize teacher noticing and decision-making processes, support adaptive teaching, foster alignment with competence frameworks, and cultivate professional vision, thereby framing teacher practices within the framework of professional vision. We argue that rather than looking at the process of adopting AI solutions by teachers from a technology perspective or how teachers contribute to designing and developing such tools, we take the perspective of the teacher and ask how such tools are meaningfully integrated into teacher practices. In our conceptual paper, we illustrate the case of a novel approach to the teacher training model where the development of teacher' professional vision and professional learning is combined with the design of the AI solutions. We argue the importance of involving teachers into the design of AI solutions through professional learning models to support teachers to develop knowledge-based reasoning skills and at the same time to learn about pedagogical concepts and develop new mental models.

## 1 Introduction and theoretical underpinnings

After more than 10 years of intensive research in the field of Learning Analytics (LA) and Artificial Intelligence (AI) in Education, there is a still a major challenge on how pedagogical practices can fully take advantage of such technologies and how those technologies could be integrated into teachers' practice (Kuhl et al., 2019). In the specific case we are investigating, which involves the usage of model-based LA tools by teachers with intelligent learning systems and the data those systems provide, there are relevant subfields within AI in education and LA. By model-based LA we mainly consider intelligent learning technologies designed to make pedagogical or instructional models transparent to teachers (Ley et al., 2023).

Such intelligent and AI-enhanced LA solutions have been developed to support or complement several teachers' practices, especially in the context of math and a variety of tools have been developed. Eduten platform provides weekly plans for the teachers and automates assessment, similarly ViLLE platform is designed to support teachers to either create or select digital content to support computational thinking skills and system supports teachers in automated assessment process. Celik et al. (2022) conducted literature overview where they argue that AI-enhanced systems are used in education mainly to decrease the teaching burden and reduce teaching load (by assisting teachers to plan interventions, assisting teachers in deciding on the learning content during lesson planning, implementation: orchestration etc., monitoring: reflection, improvement). The emphasis on professional development, training, and support for teachers using these advanced technologies remains limited in discussions (e.g., van Leeuwen et al., 2014; Rienties and Toetenel, 2016). For example, after using the ViLLE platform over time, it would be important to explore how teachers' comprehension of strategies to enhance computational thinking skills evolves. The current focus in AI-enhanced systems is to deliver technologies to teachers with the aim to support those who are struggling with classroom management and content delivery. Despite the substantial body of research and the importance of Human-centered Learning Analytics, it appears that the primary focus of LA research is understanding which solutions are meaningful for teachers, supporting their ability to act and make evidence-informed decisions. In other words, the emphasis is on delivering technologies, not on fostering the development of teachers' professional competence through the interactions with AI technologies. The design of technical solutions or practices doesn't guarantee efficient uptake, as indicated also by van Leeuwen et al. (2022) and human actions within broader systems are just as important as technical advancements.

In parallel with advancements in the field of AI, broader transformations in education are taking place—international and national educational policies and visions are increasingly focusing on fostering a more personalized and student-centered educational system (Howells, 2018). The learning process is expected to concentrate on developing students' self-regulated learning skills, enhancing students' problem-solving abilities, collaborative skills, and so forth, which requires a high level of pedagogical mastery on the part of the teacher in order to plan, implement and monitor the results of the learning process. And to further complicate this topic, all states have professional standards for teachers, which define the necessary knowledge, skills and attitudes that teachers must possess.

To effectively employ intelligent tools, teachers must perceive their meaningfulness, connect them with their professional development, understand the tool's functionalities and underlying pedagogical models and trust their outputs. Instead of focusing on the technology perspective or its design and impact on teachers or students, our lens is on how teachers meaningfully incorporate these tools into their practices and also learn from it. The challenge lies in the complexity of balancing: designing student-centric lessons, integrating technology, interacting with AI, monitoring student progress, and making informed decisions. This complexity involves mastering multiple concepts, applying them, referencing pedagogical principles, and understanding student-specific implications. Reconsidering our initial argument about the role of AI in teacher professional development, we believe AI offers substantial potential to synergize teacher noticing and decision-making, aiding adaptive teaching and aligning practices with broader competency frameworks to enhance professional vision.

Given these limitations in the current discourse on teachers and AI, the current paper undertakes to model the interaction of teacher and AI-enhanced technologies with the framework of professional vision to frame teacher's practices. We propose a research-teacher partnership that aims to bring together elements of teachers' pedagogical and content knowledge and the design of AI-enhanced tools. The concept of collaboration in design has been previously articulated by several authors and further elaborated in the recent study of Campos et al. (2023) and emphasing the need to bridge the cultural context in the design process, but we suggest to go a bit further and we aim to shed light on the potential benefits of integrating AI technologies into teacher professional learning experiences, emphasizing how AI can support teachers in their decision-making processes, facilitate alignment with competency frameworks, and ultimately enhance their professional vision.

### 1.1 Teacher professional vision and knowledge-based reasoning in AI-enhanced learning environment

AI tools usually attempt to contribute to or even take over some of the typical teacher tasks such as planning learning activities or to monitor how students are engaged (e.g., van Leeuwen et al., 2023). While we argue that the range of skills teachers need to apply on a day-to-day basis is much more diverse and therefore the potential of AI is perhaps underestimated. On a daily basis, teachers are expected to plan the learning activities based on the curriculum, choose materials, deliver the content, orchestrate classroom activities, observe how students are engaged in the learning process, and respond to student actions, assess and diagnose—abilities that take time to develop and refine. Such a highest level of professional competence is often called **professional vision** and is essential for achieving high levels of teaching quality (Gegenfurtner et al., 2020), able teachers to notice information in class and engage in knowledge-based reasoning about the noticed information (Van Es and Sherin, 2002; Sherin and van Es, 2005). The concept of professional vision is proposed based on the work of Goodwin in 1990s, who initially did not introduce the concept in the context of teacher professional learning, but the concept has gained a lot of attention in the community of education through the work of Van Es and Sherin (2002, 2008) and Sherin and van Es (2005), also among others as Seidel and Stürmer (2014), Gegenfurtner (2020), and Muhonen et al. (2021).

Seidel and Stürmer (2014) proposed to differentiate noticing and reasoning as two components of teachers' professional vision. **Noticing** is the act of selectively attending to information in classroom situations (Van Es and Sherin, 2008; Schack et al., 2017) while **reasoning** is the act of interpreting noticed information based on knowledge (knowledge-based reasoning). In their framework, Seidel and Stürmer (2014) modeled **knowledge-based reasoning** (three dimensional structure) as a set of three interrelated processes: **description, explanation, and prediction**. *Description* refers to articulation of a certain classroom level situation without additional explanations (e.g., teachers' description of students working together on a task). *Explanation* refers to verbalizing interpretations of the selected information and represents the meaning making of a classroom situation by combining classroom situational context and professional knowledge of the teacher. In this phase, teachers are developing new mental models, which integrate domain knowledge, pedagogical knowledge on certain pedagogical situations. In the explanation phase, teachers are able to argue for instance why some of the problem-solving strategies to solve ill-structured tasks in physics are less effective than others or what are the requirements to solve complex problems effectively. *Prediction* refers to future-oriented consequences of the explained classroom situations toward possible actions that might unfold after the observed scene. For example, teachers can predict what it will mean in the future if students do not perform as expected on certain types of tasks.

Earlier research has shown that **cues** become quite central to support noticing practices of teachers. Cues can be either diagnostic or non-diagnostic and according to van Leeuwen et al. (2023), they can be linked with the *tasks* that students are engaging; *students* themselves; or other *contextual aspects* of the learning situation, but in any case the way teacher interprets the cues is important, because based on that teacher will arrive to diagnostic judgment and knowledge-based reasoning. It is worth investigating the cues that the teacher is paying attention to and why some of the cues are more meaningful than others, because this helps to understand where teachers are struggling the most. Research has shown (Gegenfurtner et al., 2020) that teacher experience plays an important role in teachers' knowledge-based reasoning and noticing of the cues—the more experienced and knowledgeable the teacher is, the deeper the teacher is engaged in knowledge-based reasoning.

**Figure 1:**
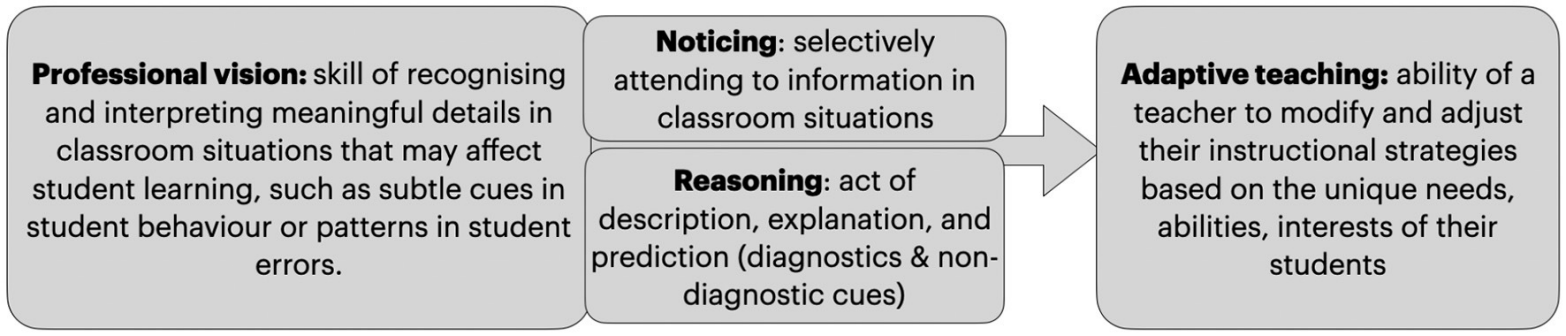
Professional vision and adaptive teaching in teacher professional development.

Higher level of knowledge-based reasoning and professional vision is tightly related to the teacher's **adaptive teaching skills**. In Figure 1, we have illustrated the relationship between those concepts: their mutual focus is on understanding and responding to the complexities of the classroom environment. Strong professional vision is a prerequisite for adaptive teaching, because teachers need to accurately interpret the learning needs and behaviors of their students, which they can only do if they have developed a keen professional vision. Adaptive teaching requires a deep understanding of the underlying concepts of effective teaching: what are different pedagogical approaches, how students' learn, what do they need to know, able to do and how to scaffold this process from designing of the tasks to use appropriate teaching methods (Brown and Campione, 1996; Darling-Hammond and Bransford, 2007; Yoon et al., 2007).

The foundation of effective teaching is built upon three key knowledge areas, as highlighted by Shulman (1987) and Borko and Putnam (1996): First, the content knowledge, which is rooted in a teacher's complex comprehension of the subject they are teaching, capturing the main principles of the domain. Second, general pedagogical knowledge, which covers an understanding of the essence of learning, the mechanisms behind it, and overarching teaching strategies applicable to any subject, such as self-regulated learning or problem-based learning. Third, pedagogical content knowledge, which centers the knowledge about how to effectively explain subject-matter to students as well as knowledge about students' potential misconceptions. These three knowledge areas contribute to the development of teachers' professional vision and are strongly shaped by the teachers' teaching practices.

### 1.2 Researcher-teacher partnership to develop teachers' professional vision through the design of AIED solutions

Gomoll et al. (2022) have proposed that development of teachers' professional vision could be promoted through the collaboration with researchers. On one hand, the development of professional vision is a complex process, requiring teachers with multi-facted competences, on the other hand such a setting provides for the researchers to learn about how to better recognize, support, and teach ambitious pedagogy (van Es et al., 2017). In this conceptual article we argue that design of LA tools and practices for the teachers could be embedded systematically into teachers' professional learning. Especially in the field of learning technologies, there has been an assumption that it is enough to introduce teachers to novel technologies and such a transmission of information helps teachers to replicate the practices that they have been exposed to. However, there is substantial evidence, which demonstrates that tools that teachers use become part of their professional practices (e.g., Wise and Jung, 2019). They shape and are shaped at the same time by these practices. One way of integrating tools into professional practice is by viewing the teacher as a reflective practitioner theoretically supported by the term of experiential learning and the work of Dewey, Piaget, Vygotsky (Girvan et al., 2016). Although the work of Kolb, who has proposed the phases of experiential learning (concrete learning, reflective observation, abstract conceptualization, and active experimentation) has been criticized a lot, the core idea is the individual level change process through the action, which results in experience, reflection on action and experience, concept drawn from reflection and action resulting from this reflection. Darling-Hammond and McLaughlin (2011) have suggested that professional development inspired by experiential learning model could motivate teachers to design and evaluate new practices and make changes in instruction. Experiential learning models are based on the constructivist theory, which suggests that learners create new knowledge and develop mental models through their own experiences, according to Evans (2019). Kolb et al. (2001) have suggested four iterative phases of experiential learning:

- In the **Concrete experience** phase, teachers encounter a new experience or reinterpret an existing experience: for example, in the case of our program, they may have carried out a problem-based learning task by experimenting with different technologies.
- **Reflective observation** phase refers to the activities where the experiences are reflected from different perspectives.
- In the **Abstract conceptualization** phase teachers make connections between what they experienced and noticed with the concepts learnt during the training.
- In the **Active Experimentation** phase the teacher validates the theories formed in the Abstract Conceptualization stage in new situations.

Throughout each of these phases, the teacher faces the challenge of understanding the theory, implementing new methodologies, and navigating the integration of technological advancements. In this type of professional training, where the integration of theory, practice, implementation, and new technologies takes place, it becomes increasingly apparent that teachers require substantial support and collaboration. Teacher-researcher partnerships provide efficient ways to address this need.

According to our knowledge, there is not enough discussion on how to integrate AI into professional learning supporting professional learning phases and taking into account a model of professional competence. Professional vision framework is a good starting point for that, because AI could support teachers' in noticing and making sense of what is going on in the classroom. However, AI has the potential to go even further and translate the cues, decisions and knowledge-based reasoning incidents into the development of teacher professional competence. For instance, in the context of Estonia, teachers' qualification standard defines one of the core competence of teachers as “Supporting students” and sub-competence may include activities as: “*The teacher is* ***aware of the foundations*** *of the learner's cognitive development; is* ***aware*** ***of the factors*** *influencing learning and of evidence-based ways of supporting the learner;* ***identifies the learner's*** *level of subject knowledge, learning skills and motivation to learn and t****akes these into account*** *when setting learning objectives;* ***recognises learners' need*** *for support and their individual learning needs;* ***supports the*** ***development of social and cooperative skills****; takes into account group processes and dynamics*”.^1^ We can see that this particular competence is a very complex one, which requires teachers to have a very high level of competence in carrying out a wide range of activities: planning, noticing, reacting, finding information, making connections, applying different strategies. It is very difficult to do all of this if the teacher's knowledge of some of these aspects is still lacking, or if he or she lacks the ability to notice.

As discussed earlier, explaining and predicting as the sub processes of professional vision have been found difficult for the teachers, because it assumes the linkage of the detected classroom situations with broader professional prospects (Muhonen et al., 2022). Clearly AI could be a supportive element in this process. However, it will be especially effective if it is combined with the teacher training. Qvortrup (2016) emphasizes the need for close research-practice relationships to drive meaningful change and improvement in pedagogical practices. One of the ways to bring together research and practice is the implementation of teacher professional development programs. Effective teacher professional training has been conceptualized through different theoretical framings from experiential to situated approaches. Borko (2004) has argued that in teachers' professional learning, it is important to focus on *subject matter knowledge for teaching*, developing *an understanding of student thinking*, and fostering the development of *instructional practices*. Several meta studies have suggested that effective teacher professional development is content focused; incorporates active learning strategies and engaging teachers into the design and implementation of new practices to make connections between professional learning and classroom situations; enhances collaboration in job-embedded contexts; integrates mentoring, fosters feedback and reflection (e.g., Darling-Hammond et al., 2017). Similar approach has been followed by Ley et al. (2022) and the results of which showed that, through co-creation in research-practice partnerships, teachers were more likely to adopt the innovation.

### 1.3 Toward human-centered design of AI and teacher partnerships

Recent literature in the field of AI and LA have emphasized the importance of human-centered approaches. For instance Tsai and Martinez-Maldonado (2022) have argued that human-centered methodologies and engagement of key stakeholders is essential in developing LA solutions and practices and recently the human-centered approach has gained a lot of attention in the LA community. There are a number of approaches to involve users in the design of learning analytics solutions and practices. Sarmiento and Wise (2022) carried out a systematic review on how participatory and co-design approaches have been implemented in the design of learning analytics solutions, methods that have shown the great potential to support the teachers' agentic behavior while interacting with LA (Tsai and Martinez-Maldonado, 2022). Techniques such as understanding end users' needs before design, testing LA solutions after development with stakeholders, generating ideas during design sessions, early evaluation and co-development have been applied in LA research on engaging stakeholders to the design of LA solutions. For instance the study of Herodotou et al. (2019) found evidence that participatory design approach to design LA dashboards increased teachers' perceived adoption. Based on Verbert et al. (2013), by LA dashboard we mean the solutions that highlight learning progress and areas of improvement for educators and students, promote awareness, reflection, and offer actionable recommendations, thereby enabling informed decision-making.

Avila et al. (2020) carried out an experimental study to understand if developed LA solution benefits teachers in the acquisition of competences in the creation and evaluation of OERs, but without interacting with OERs in real authentic settings with the possibility to observe the effect of OERs on students' learning. These are just a few examples of possible ways to take teachers' voice into account in the design of LA or AIED solutions in education, which still assume that the teachers are “used” as a way to design more appropriate tools. However, this underestimates the fact that design is also a way to change teaching practices, which we will elaborate in the next paragraph.

Recently, researchers have argued that a participatory approach is not enough. Dimitriadis et al. (2021) argued that in the process of human-centered LA it is important to promote the agentic position of teachers, integrate of the learning design cycle and the design process and reliance on educational theories to guide the LA solution design and implementation. van Leeuwen et al. (2023) went even deeper and involved teachers not only in the design of dashboards, which are theory-based, but teachers were also involved in the development of theory-led cues, contributing to the development of the teacher's professional vision. It can be argued that these types of methodological approaches not only help to create pedagogically sound solutions, but also thereby support the uptake of solutions. Vatrapu et al. (2011) introduced already more than 10 years ago a theoretical approach combined dashboard, teaching expertise and design-based research in a triadic model to support teacher's diagnostic pedagogical decision-making in classrooms. Co-designed dashboards could be turned to usable and effective for the teachers' professional learning, but only a dashboard is not enough, there is a need for teacher training to reach the full potential of such dashboards (van Leeuwen et al., 2023), because the design of any technical solutions or practices does not ensure efficient uptake and human actions in wider systems are as important as technical advancements (van Leeuwen et al., 2022).

Despite advances in the integration of AI in education, we have an incomplete understanding of when and how teachers construct new knowledge and mental models while interacting with AI solutions. A potential reason is that teachers are often involved in studies for only brief periods, leaving insufficient time to observe the critical changes in their understanding. There's a need to fully understand teachers' development in terms of their professional vision, the integration of higher-order pedagogical concepts, innovative teaching methods, and comprehending these concepts in practical scenarios concerning effective learning and strategies. To address this gap, we propose an emphasis on the long-term and intensive engagement of teachers through dedicated professional development programs. Furthermore, establishing research-practice partnerships can offer continuous feedback and a deeper understanding of the teachers' journey in adopting and adapting to AI solutions.

## 2 A model and illustrative case on integrating the design of AI solutions and teacher professional competence

### 2.1 Conceptualization of teacher PD to design model-based LA solutions

**Figure 2:**
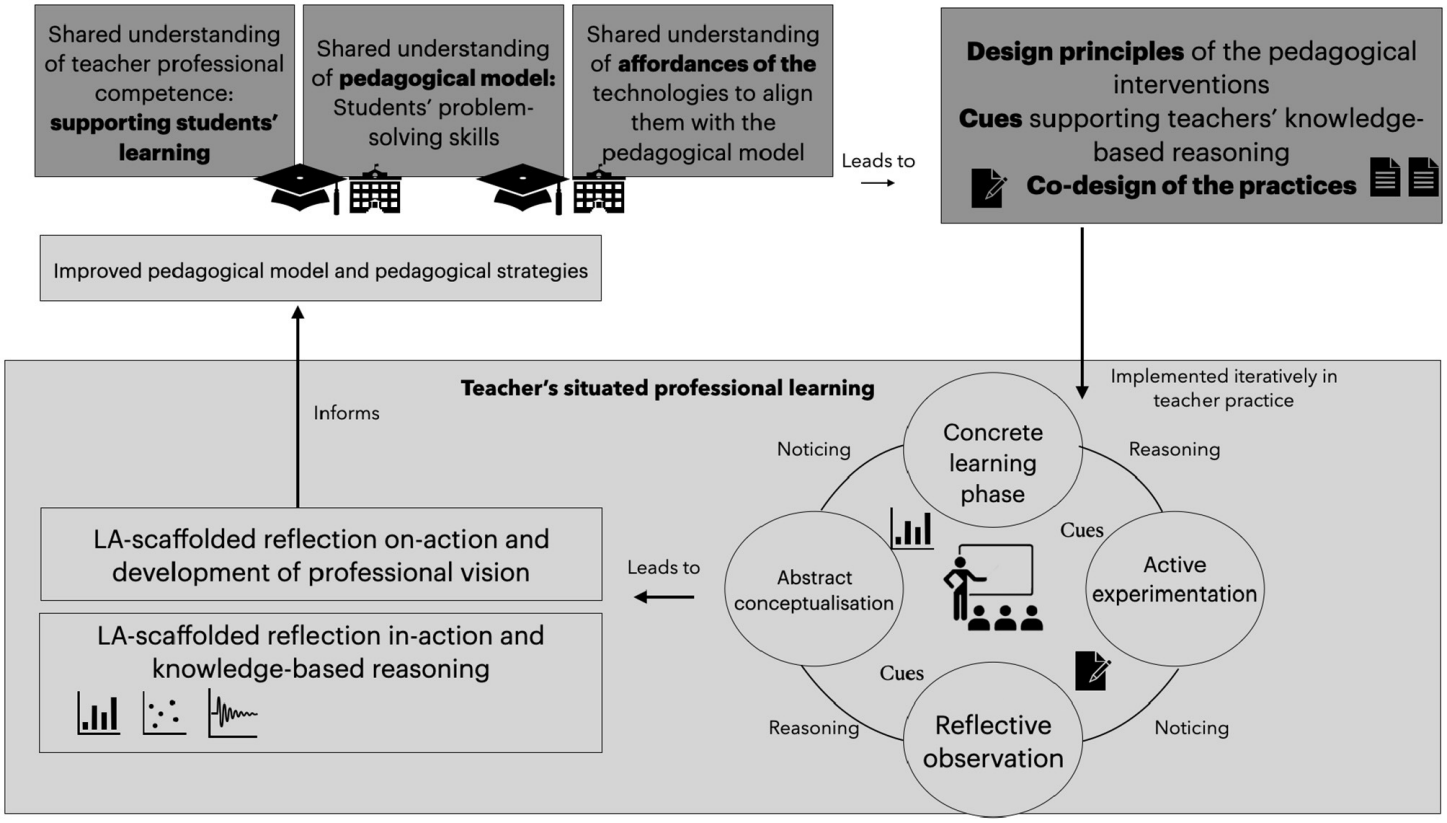
Conceptual model on integration of three elements: teacher training, design of AI solutions, and development of teacher professional vision through research-practice partnerships.

In this chapter we propose the conceptual model for teacher professional development, seamlessly blends individual professional development for teachers with collaborative learning experiences, incorporating both elements across various stages of the intervention. The model aims to conceptualize how to relate dashboard use and design with professional learning models, to ensure that LADs meet the needs of teachers' professional practice and its usage could lead to the development of teachers' professional vision (see Figure 2). Such an approach would enable teachers to build ownership with the technology, understand the data, models and decisions behind the tool and at the same time to learn how to design pedagogical interventions, which are informed by the dashboard.

The conceptual model has resulted from a synthesis of some of the prior literature mentioned in the previous section, as well as our previous theoretical and empirical work. First, we framed the concept of knowledge appropriation within teacher-researcher partnerships when teachers adopt educational innovations by drawing on Ley et al. (2022). Second, the model was elaborated into a training intervention called Teacher Innovation Laboratory which should foster important practices of Knowledge Appropriation (Leoste et al., 2019). Third, the principles of teacher professional learning in the Digital Learning Ecosystem (Tammets et al., 2022) were integrated into the model to highlight the importance of holistic professional learning experiences supported by learning technologies. Finally, we integrated elements of the work of van Leeuwen et al. (2023) highlighting the importance of a participatory approach and integration of teachers into the design of theory-grounded dashboard to support the development of professional vision. Several of the individual elements of this model have been informed by our active work with teachers and teacher trainers in Estonian secondary schools. For example, the Teacher Innovation Laboratory has been completed by over 150 teachers in all subject areas who are integrating digital technology into their teaching. The illustrative example that is given below describes experiences of one of those teacher groups. In subsequent research, we will empirically evaluate the impact of the model on the enhancement of teachers' professional vision.

The upper part of Figure 2 illustrates the importance of **research and practice partnership**, which forms the basis to build a shared understanding of teacher professional competence, pedagogical models and affordances of the learning technologies. In this setting, social learning practices and collaborative learning design process between teachers and teacher educators and researchers is fostered. Through the collaborative sessions, researchers and teacher educators blend the pedagogical and domain knowledge with the possibilities of learning technologies and jointly the design principles will be formed as also the important cues that teachers should notice in the classroom while implementing new practices. This phase is important to build the shared storing pedagogical foundation among the teachers, but also to create guidelines and models that teachers' can adapt for their practice.

Lower part of the drawing conceptualizes **teachers' iterative situated professional learning process**, which integrates individual and collaborative learning. By integrating the design principles, good practices and co-created pedagogical practices, teachers need to embeds them into their own practice. Such an iterative process will happen several times during the PD and is framed by the phases of **experiential learning** (Kolb et al., 2001):

- During the **concrete experience** phase, teachers directly engage with students' in concrete lessons, implementing learning technologies that collect data on student performance. In this process, teachers gather valuable insights into their current strategies, observe students' reactions, and gauge their own awareness of the students' learning processes. Learning technologies may provide instant feedback for the teachers to support the reflection in-action and to adjust the instructional approach in real time. However, technologies may collect the data and inform the teacher later to scaffold the reflection on-action process in the training session.
- The **reflective observation** phase involves engaging in activities that encourage teachers to reflect on their experiences from various perspectives, discuss relevant cues, and develop the ability to notice and reason about classroom situations. For instance, a teacher uses intelligent learning technologies (e.g., Digital Learning Resources giving feedback about students' learning) to observe patterns in students' performance and engagement. Reflecting on the data, a teacher is able to identify common areas of struggle and varying engagement levels throughout the learning activity, preparing her for the next stage of conceptualizing instructional adjustments. During this phase, the data collected from the classroom becomes crucial as it facilitates the reflection process.
- During the **abstract conceptualization** phase, teachers establish connections between their classroom experiences, observations from the reflection phase, and the concepts they have learned during their training sessions in researcher-teacher partnership. This phase involves designing lessons, materials, tasks with intelligent learning technologies based on the domain model. Concurrently, this phase includes the integration of AI solutions to seamlessly connect theoretical knowledge with practical application. Teachers actively participate in the design process led by the trainers and researchers creating links between pedagogical materials (lesson plans, learning materials), domain models, and AI solutions. In this phase the co-design of the feedback loops, cues, scaffolding elements will take place.
- In the **Active Experimentation** phase the teachers validate the theories formed in the Abstract Conceptualization stage in new situations in their own practice with their own students.

Intelligent learning technologies become central in this process: teachers integrate pedagogical knowledge into the affordances of the tools: for instance teachers are trained to understand the importance of activating prior knowledge in complex problem-solving tasks, what are the strategies to activate the prior knowledge, how different tools can be used for this purpose. But not only—teachers are integrated into the design of LA dashboards which help them to understand to what extent students activated prior knowledge and what it means if some students had no prior knowledge. In practice, during the training program, the dashboard serves as a bridge between theoretical training and hands-on application, aiding teachers in the reflection and abstract conceptualization stages. It enables them to draw connections between theory and actual practice, identify key cues, and gain insights into students' learning processes. The LA dashboard plays a central role in transferring cues from the training context to real-world practice. This not only strengthens teaching methodologies but also fosters the transfer of training knowledge. Such a professional training program, underpinned by a teacher-researcher partnership, facilitates the refinement of pedagogical models integral to AI solutions. Over time, these structured and scaffolded processes are poised to enhance teachers' professional vision and their ability to adapt teaching techniques.

### 2.2 The example case: mathematics teachers' to support students' learning in problem-solving scenarios

In this section we describe the case to illustrate how our conceptual framework has been implemented. Twenty-six primary school mathematics teachers participated in the 9-months training. The aim of the PD intervention was to support teachers to understand how to design learning scenarios, activities and digital learning resources based to support students' learning in problem-solving scenarios. During the PD program, teachers learnt what is problem-solving in math and what are the underlying concepts. They designed new practices around learning technologies, implemented those iteratively in the classroom, gathered data and participated in collective reflection to share the experience and introduce improvements.

**Figure 3:**
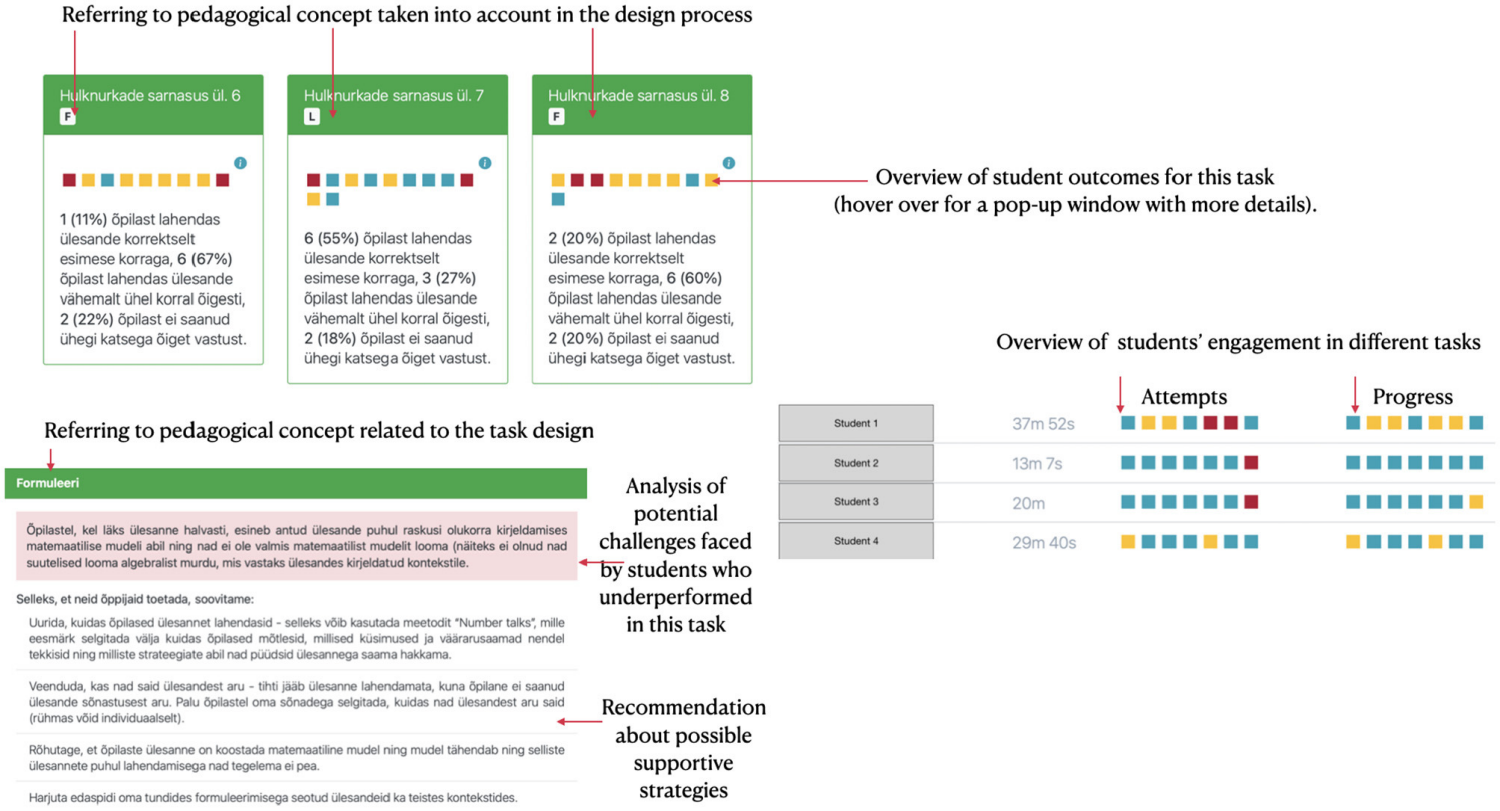
Dashboard views co-created with the teachers.

During the PD, **monthly sessions** were conducted to foster teachers' mutual understanding of students' mathematical problem-solving process. These sessions explored the requisite teacher knowledge and skills, including necessary knowledge, design requirements, supportive strategies, and observational cues. Additionally, design principles for creating scenarios in digital learning environments were discussed. Teachers actively participated in these sessions, co-designing pedagogical practices and digital learning resources and tasks based on established guidelines. The content was created using H5P templates, while a Drupal-based authoring tool provided teachers with a platform to design educational materials rooted in pedagogical principles. Furthermore, teachers were deeply involved in the iterative design of LA tools and practices. Separate module was designed during the training to provide model-based feedback for the teachers about the implementation of their H5P-based tasks in their own classrooms. Feedback was mainly designed to offer teachers insights into the effectiveness of their applied strategies and to identify possible reasons why some of the students underperformed. This was realized by LA dashboards that teachers could use to analyze as illustrated on Figure 3. Figure illustrates the various perspectives teachers engaged with in the dashboard design. The figure's left corner presents a task-oriented summary: tasks created by the teacher, aligned with specific pedagogical concepts from the training, show an aggregate student progression. It was anticipated that students would be more adept at L-type tasks, evident from more blue dots indicating first-attempt success. The right corner displays individual student progress across tasks of varying complexity (details available on hover). Beneath this, feedback for each task type outlines potential student challenges and suggests strategies for future support. The primary teacher involvement in design was centered around the task view, aligning with the professional development's focus.

**Figure 4:**
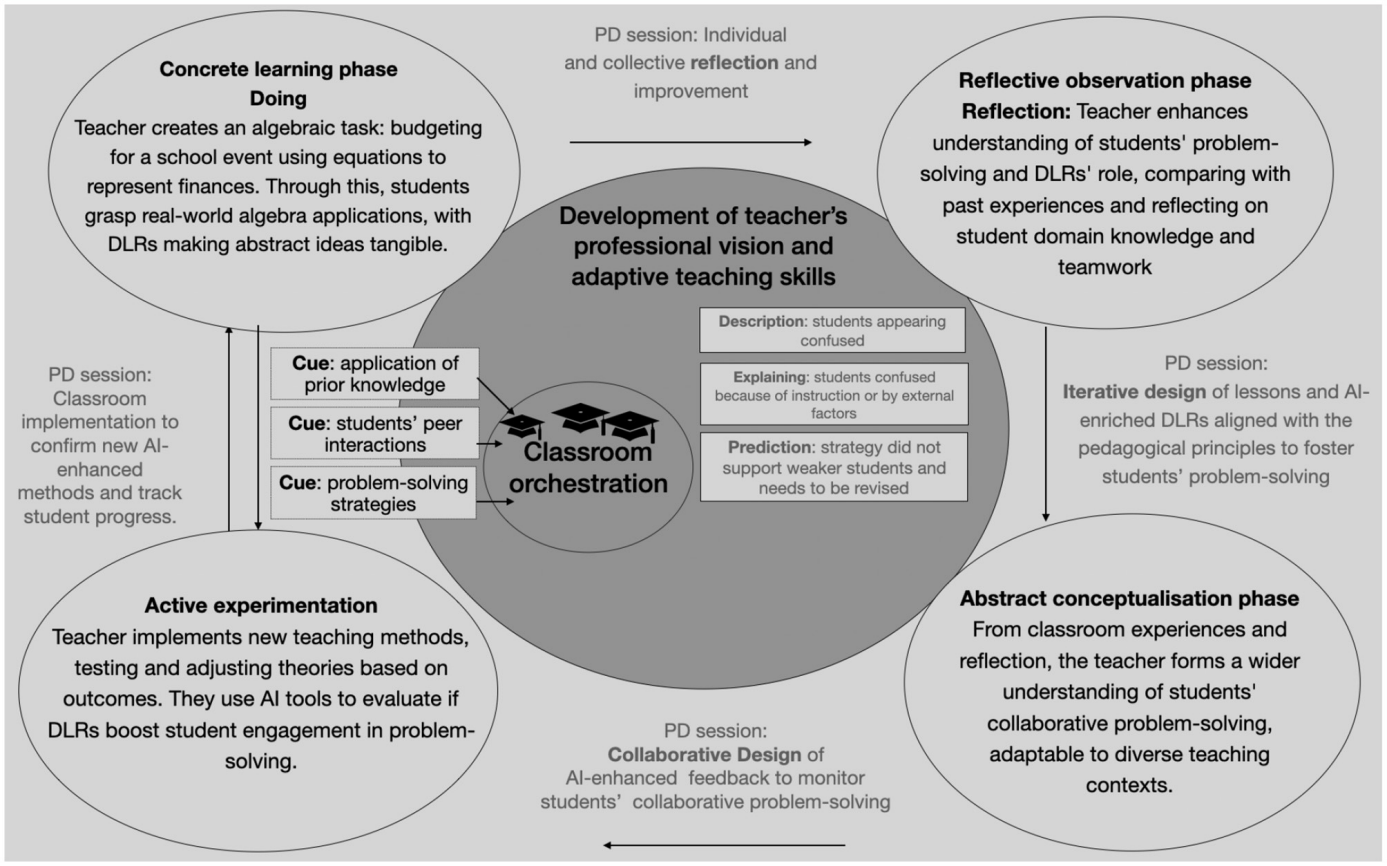
Enhancing teacher's professional vision through experiential learning in a researcher-teacher partnership.

Figure 4 illustrates how the phases of experiential learning were actualized through a researcher-teacher collaboration in this concrete case. The phases of ***Reflective observation*** and ***abstract conceptualization*** emphasize collaborative interactions, fostering a shared understanding and synergizing experiences, pedagogical concepts and AI-enhanced tools. ***Active experimentation*** and the ***Concrete learning*** phase are interconnected, with one setting the foundation for the other. Those two phases highlight the importance of teacher practice and reflectiveness, which are iteratively promoted during the PD. In this process, teachers are taught to notice cues related to the pedagogical concepts by using AI-enhanced tools.

Dashboards that were developed, enabled teachers at the end of the training to analyze their students' progress and notice certain cues based on the guidelines of the researchers. After each iteration, collective reflection took place as part of the training. Teachers were encouraged to make connections between the learnt concepts (complex problem-solving in mathematics), create lesson plans and materials, students' interactions and LA tools' visualization. By evaluating classroom data through the lens of pedagogical concepts, students' learning, and domain models, they were able to perceive the effectiveness of the LA tools. Such an approach enables teachers to on one hand link the training and practice, but on the other hand build an understanding on the link between pedagogical and domain knowledge and students' interactions.

In the middle of Figure 4 two darker gray circles are presented related to teacher professional vision and teaching skills. The smaller circle represents everyday classroom orchestration and management of the teachers in the active experimentation and learning phase supported by the AI-enhanced tools: for instance students' results can indicate their usage of effective problem-solving strategies, their task results could indicate insufficient prior knowledge and their collaborative actions may indicate that not all the students are engaged. The bigger circle represents the wider teacher professional learning aspect. AI-driven learning tools can aid teachers in enhancing knowledge-based reasoning. Using theory-informed dashboards, teachers can monitor student engagement, pinpoint learning gaps, and adjust their methods instantly. This constant feedback refines their understanding and ability to predict student responses to different strategies. Such capabilities indicate not only improved teaching competencies but also the effective transfer of knowledge from professional training to the classroom, signifying holistic professional development (Stürmer et al., 2013).

AI-enhanced tools have the capability to not only provide insights but also to integrate cues, decisions, and knowledge-based reasoning into the framework of the official occupational qualification standard of teachers. For a competency-focused approach to teacher professional development, this integration is crucial. Taking the Estonian Teacher Professional Standard as a reference, which stresses conscious support for students, the PD program ensures teachers grasp the nuances of learner cognitive development, especially in problem-solving contexts. AI-enhanced tools augment this grasp by helping teachers perceive students' baseline knowledge essential for efficient problem-solving. They then integrate this understanding into instructional planning. As lessons progress, AI-enhanced tools help spotlight student needs and in real-time, tools allow flexible teaching to adjust according to dynamic student needs. With sustained training and AI tool usage, teachers gain deeper insights into student problem-solving ability and strategies, refining own professional vision. Such a refined vision invariably sharpens teaching practices, fostering enhanced professional growth.

## 3 Conclusion

In our conceptual paper, we presented a case on how to integrate the triangle of teacher PD programs, design of teacher AI solutions and long-term development of teacher professional vision with the aim to enhance the adaptive teaching skills of teachers. We demonstrated the potential of AI solutions to support teachers to develop knowledge-based reasoning skills and at the same time to learn about pedagogical concepts and develop new mental models. Currently the research in LA and AI and teacher adoption is focused either on classroom orchestration level or teacher PD, looking at these aspects in separate ways, but in our approach it is important to look at them together. We propose that integrating teacher training into this formula contributes to more evidence-informed teaching practices and development of teachers skillset. Dashboard design that would play a bridging role between the training context and the practical context by moving some of the cues from training to practice. This could enforce the practices and contribute to transfer of training.

This is the first phase of our design study where we have created and implemented this concept of teacher training to design AI solutions. The next step is to find answers to the research questions that we believe could help the research community to understand how such approaches could lead to better partnerships between teachers and AI, support teacher adoption of AI and lead to changes in teacher practice. It would be important to understand what are the implications for the design and delivery of such programs, potential challenges and benefits of integrating AI technologies into teacher professional learning experiences, as perceived by teachers? In addition, we see this context as an opportunity to investigate how we can effectively monitor learning moments within teachers' professional development. Specifically, the aim could be to examine which cues hold significance and when, and how we can enhance our models and AI solutions based on the insights gained from these experiences. It is also important to think about how to methodologically evaluate teacher learning and adoption, in order to gain as comprehensive a picture as possible of the development of teachers' professional vision and adaptive teaching skills.

## Author contributions

KT: Conceptualization, Writing—original draft. TL: Conceptualization, Writing—review & editing.

## Footnotes

^1^ https://www.kutseregister.ee/ctrl/et/Standardid/vaata/10824233
